# Monitoring of the lactonase activity of paraoxonase-1 enzyme in HIV-1-infection

**DOI:** 10.7448/IAS.17.4.19682

**Published:** 2014-11-02

**Authors:** Clara Dias, Aline Marinho, Judit Morello, Gabriela Almeida, Umbelina Caixas, Karina Soto, Emilia Monteiro, Sofia Pereira

**Affiliations:** 1 Faculdade de Ciências Médicas, Centro de Estudos de Doenças Crónicas (CEDOC), Universidade Nova de Lisboa, Pharmacology, Lisboa, Portugal; 2 Departamento de Química, Instituto Superior de Ciências de Saúde Egas Moniz, REQUIMTE, CQFB, FCT-UNL, Caparica, Portugal; 3 CEDOC – Pharmacology, Centro Hospitalar de Lisboa Central (CHLC), EPE, Lisboa, Portugal; 4 CEDOC – Pharmacology, Hospital Prof. Doutor Fernando Fonseca (HFF), EPE, Amadora, Portugal

## Abstract

Paraoxonase-1 (PON1) is a high-density lipoprotein (HDL)-associated enzyme known as a free radical scavenging system (1). PON-1 has three main activities, responsible for its antioxidant and anti-inflammatory potential: paraoxonase, arylesterase and lactonase (LACase), the latest to be discovered and pointed out to be its native activity (2). Among other physiological roles, the LACase might minimize the deleterious effects of hyperhomocysteinaemia in infection, by detoxifying the highly reactive metabolite homocysteine-thiolactone (HcyTL) (3),^4^. In the present work, we have developed and applied a method to quantify LACase activity and to explore the role of this enzyme in HIV-infection and virological response. The LACase activity was monitored in a cohort of HIV-1-infected patients, through the titration of 3-(o-hydroxyphenyl) propionic acid, formed upon the LACase-mediated hydrolysis of the substrate dihydrocoumarin. The study protocol was approved by the Ethics Committee of Centro Hospitalar de Lisboa Central and Hospital Prof. Doutor Fernando Fonseca. All patients gave their written informed consent and were adults with documented HIV-1-infection, regardless of combined antiretroviral therapy (cART) use. Naïve patients and patients who had received continuous antiretroviral treatment for more than one month were included. A total of 179 HIV-1-infected patients were included on this study (51% Men, 39% non-Caucasian, 45±13 years old). Patients with non-suppressed viraemia, either from the non-cART (n=89, 12±4 kU/L, p<0.01) or from the cART with detectable viral load (n=11, 10±5 kU/L, p<0.05) groups, had lower activity than the cART with suppressed viraemia (n=79, 15±7 kU/L) (Kruskal–Wallis test). Among naïve patients, higher viral load (> 31,500 cps/mL, Spearman r=−0.535, p=0.003) and lower CD4+ T-cells count (< 500 cell/mm^3^, Pearson r=0.326, p=0.024) were associated with the LACase activity. The present study suggests that lower LACase activity is associated with uncontrolled HIV-1-infection, particularly with non-suppressed viraemia, despite of cART. This data seems to point to LACase role in HIV-infection, probably reflecting an increased formation of HcyTL deleterious species. A better knowledge of the LACase and its role in HcyTL pathophysiology might identify new therapeutic targets in HIV-1-infected patients.

**Acknowledgements:**

EXPL/DTP-FTO/0204/2012; EXPL/DTP-PIC/1758/2013.
